# A Discrete Fruit Fly Optimization Algorithm for the Traveling Salesman Problem

**DOI:** 10.1371/journal.pone.0165804

**Published:** 2016-11-03

**Authors:** Zi-bin Jiang, Qiong Yang

**Affiliations:** College of Business Administration, Hunan University, Changsha, Hunan, China; Beihang University, CHINA

## Abstract

The fruit fly optimization algorithm (FOA) is a newly developed bio-inspired algorithm. The continuous variant version of FOA has been proven to be a powerful evolutionary approach to determining the optima of a numerical function on a continuous definition domain. In this study, a discrete FOA (DFOA) is developed and applied to the traveling salesman problem (TSP), a common combinatorial problem. In the DFOA, the TSP tour is represented by an ordering of city indices, and the bio-inspired meta-heuristic search processes are executed with two elaborately designed main procedures: the smelling and tasting processes. In the smelling process, an effective crossover operator is used by the fruit fly group to search for the neighbors of the best-known swarm location. During the tasting process, an edge intersection elimination (EXE) operator is designed to improve the neighbors of the non-optimum food location in order to enhance the exploration performance of the DFOA. In addition, benchmark instances from the TSPLIB are classified in order to test the searching ability of the proposed algorithm. Furthermore, the effectiveness of the proposed DFOA is compared to that of other meta-heuristic algorithms. The results indicate that the proposed DFOA can be effectively used to solve TSPs, especially large-scale problems.

## Introduction

The traveling salesman problem (TSP), one of the most complex combinatorial optimization problems, has been extensively studied due to its practical applications. This problem can be described as a salesman who wants to travel a series of *n* cities. Suppose that *d*_*ij*_ (*i*,*j* ∈ {1,2,⋯,*n*}), which denotes the distance between the traveling points *i* and *j*, is well known by the salesman. The salesman wants to select the route or tour that includes one stop in all of the cities with the minimum travel distance. The route can begin in any city, but the salesman must return to the city of departure. Other factors, such as time and cost, can be considered as well. In a TSP, if the travel distance or cost from city *i* to city *j* equals from *j* to *i*, then it is considered to be a symmetric problem, or otherwise, an asymmetric problem. Since any asymmetric Euclidean TSP can be transformed into a symmetric problem, symmetric problems have been more extensively studied. In fact, symmetric problems, particularly Euclidean TSPs involving cities located in a two dimensional plane and in which Euclidean distances are used as a metric, play an important role in practical applications, such as VLSI chip fabrication, X-ray crystallography, flexible flow shop scheduling, and schoolbus routing [1,2]. However, the TSP has been proven to be an NP-hard problem, in which any exact approaches to determining optimal solutions may necessitate the long running times associated with high dimensionality [3]. As a result, researchers have primarily developed approaches that can only obtain near-optimal solutions in a relatively short running time. However, others have attempted to develop optimization algorithms that function substantially well in practical cases rather than worst-case scenarios. Due to the importance of this problem in both practical applications and academic research, intelligent and knowledge-based algorithms are needed.

In the past twenty years, TSP problems have served as benchmarking and initial testing tools for novel algorithms. These algorithms can be classified as exact approaches and approximate approaches or heuristics. Exact approaches are used to enumerate the optimal tours of finite-stage TSPs. However, the running times of exact approaches are comparatively long in power time complexity. Therefore, exact approaches cannot be effectively applied to large-scale problems. In contrast, heuristics can be used to determine good tours in polynomial time complexity, although they do not necessarily yield the optimum tours. Seyed Mohsen Mousavi et al. have developed two parameter-tuned meta-heuristics and two meta-heuristics algorithms to solve a discounted inventory control problem or multi-item multi-period inventory control problem under storage constraints and discounts [4,5], which greatly increased the scope of application for heuristics. The heuristics for TSP can be subdivided into travel cycle construction approaches and improvement approaches. In travel cycle construction approaches, a tour is generated in *n* steps by gradually adding the indices of different cities. Strategies used to select the city in the next step include the nearest or farthest neighbor criteria, greedy method, Clarke-Wright algorithm [6], and Christofides algorithm [2]. In tour improvement approaches, an entire tour is generated. Then, improvement or exchange strategies are employed to improve those tours. These strategies include local search or local optimization, simulated annealing [7], ant colony optimization [8], particle swarm optimization (PSO) [9], and genetic algorithms (GA). Especially, PSO has been improved and used in many fields to solve problems like a multi-product multi-period inventory control under inflation and discount [10], and the integrated location and inventory control in a two-echelon supply chain network [11]. However, local search is the most simple and effective approach. Since most of the earlier studies that used GA to solve TSP focused on designing proper encoding representations, reproduction, crossover and mutation operators [12–14], they followed the evolution strategy of simple genetic algorithms. Although these methods can be used as valuable references for other evolutionary optimization algorithms, their performances are lacking compared to local search approaches, such as the two-OPT, three-OPT, and Lin-Kernighan (LK) approaches [15]. Currently, other new evolutionary optimization algorithms for TSPs exist, such as the discrete bat algorithm [16], discrete firefly algorithms [17], and the discrete invasive weed optimization algorithm [18]. Although these algorithms have yielded good results, they still require further improvements and modification. Usually, tour construction and improvement are combined to allow for the construction of initial tours and later improvement of those tours, respectively. Therefore, a hybrid approach is more applicable. Baraglia *et al*. developed a hybrid GA with LK local search capabilities [19], Hung *et al*. also developed a hybrid GA with LK in order to improve the local search capability of the GA [20]. Hybrid GAs utilize global optimization capabilities of a GA and local optimization capabilities of other heuristics to overcome premature. However, due to the complexity of genetic operators and local improvement operators, hybrid GA approaches entail high computational loads and long CPU running times. Thus, these approaches are not best applied to large-scale problems.

Recently, Pan developed a novel optimization algorithm called the fruit fly optimization algorithm (FOA) based on swarm intelligence by carefully observing the foraging behavior of fruit flies [21]. The FOA possesses numerous advantages, including a simple structure, few adjustable parameters, and a relatively short CPU running time. The FOA is also easy to program and can be modified to other practical applications. Due to these advantages, the FOA has been used to solve a wide range of optimization problems, including prediction and classification problems [22–24], continuous function optimization problems [25], the multidimensional knapsack problem [26], and scheduling problems [27]. And in the newest research fields, identification of dynamic protein complexes [28], selecting evolutionary direction intelligently and joint replenishment problems [29,30], also can acquire good results based on fruit fly optimization algorithm. Although the original FOA has primarily been applied to problems on a continuous definition domain, it can also be successfully applied to problems with continuous variables. However, the FOA must be modified in order to effectively manage the discrete variables associated with combinatorial optimization issues, such as the food source representations and effective generation mechanisms of candidate solutions near swarm locations in the TSP, intelligent parallel test sheet generation [31], and flow shop scheduling problems with intermingling equivalent sublots [32], optimizing a location allocation-inventory problem in a two-echelon supply chain network [33], and the homogeneous fuzzy series-parallel redundancy allocation problem [34]. As stated previously, the TSP is an NP-hard combinatorial optimization issue involving a large search area that cannot be easily solved with traditional algorithms. However, the FOA is a parallel evolutionary algorithm based on smelling and vision processes. In addition, problem-dependent operators can be modified to adapt the smelling process of an FOA to further enhance exploitation. Furthermore, local search methods can be effectively incorporated by sharing information regarding swarm food locations to precipitate exploration. Therefore, the FOA could be modified to solve TSP. Li Heng-yu adopted and adapted step size and mutation strategies in order to solve TSPs [35]. Wang Ke-fu *et al*. introduced the radius of local optimum through which whether the fruit fly was in a local optimum area could be judged [36]. Roulette method is used to initialize the path. At the same time the local search ability and convergence speed up by using C2Opt to optimize the local path [37]. Yin Lvjiang *et al*. integrated PSO and GA algorithm into FOA to improve its advantages. IFOA and PSO were compared; for most of the traveling salesman problem, the effect of the IFOA is better than the PSO, different from this paper that it for all [38]. The smell function took into account randomly generated variant of the fruit fly encoding with Bit Mutation Operator, and the data sets used in the experiment were different from us. The methods have been discussed by Nitin S. Choubey *et al*. only applied to the instances with a small number of cities [39]. However, these strategies can be further optimized. Therefore, in this study, a discrete FOA containing a new strategy is used to solve the TSP. Specifically, an ordering of city indices is used to directly represent the solution. Based on the characteristics of the problem, an effective crossover operator is designed for the smelling process of fruit flies. In addition, an effective local search method, the edge intersection elimination method, is used by the fruit fly group to sense the smell of the swarm food location. According to the results, the combination of the crossover operation and edge intersection elimination strategies in the DFOA effectively prevented the occurrence of local optima and low convergence. Furthermore, the effectiveness of the proposed DFOA is demonstrated with computational tests using benchmarking problems and a comparative analysis of other nature-inspired algorithms.

The remainder of this paper is organized as follows. In section 2, the mathematical formulation of the TSP is described. In section 3, the original FOA is presented, and the DFOA procedure of the TSP is illustrated in detail. In section 4, numerical testing results obtained using a classified set of benchmarking problems and a comparison of the proposed DFOA and other existing algorithms are provided. The conclusions of this paper and future research opportunities are presented in section 5.

## Problem Description

A symmetric TSP can be demonstrated on a complete undirected graph *G* = (*V*,*E*), where *V* = {1,…, *n*} denotes a vertex set, and *E* = {(*i*, *j*) | *i*, *j*∈*V*,*i* ≠ *j*} denotes an edge set [40,41]. Suppose that the coordinates of vertex set *V* are known and that a distance matrix *D* = (*d*_*ij*_) is defined by edge set *E*. Let *y*_*ij*_ be a decision variable associated with each edge (*i*,*j*), where the decision variable *y*_*ij*_ = 1, the route from city indices *i* to *j*, represents the path selected by a salesman, while *y*_*ij*_ = 0 represents the path not selected by the salesman. In addition, suppose that *S* is the proper subset of *V*, and |*S*| denotes the number of vertices included in the set *S*. These notations and indices can be used to formulate the mixed integer programming formulation of a TSP as follows:

$$\min Z=\sum_{i=1}^{n}\sum_{j=1}^{n}d_{ij}y_{ij}$$ (1)

$$\begin{array}{l} s.t.\{\begin{array}{llllll} \sum_{j=1}^{n}y_{ij}=1, & & i\in V, & & & \left(2\right)\\ \sum_{i=1}^{n}y_{ij}=1, & & j\in V, & & & \left(3\right)\\ \sum_{i\in S}\sum_{j\in S}y_{ij}\leq |S|-1 & & \forall S\subset V, & |S|\neq \emptyset & & \left(4\right)\\ y_{ij}\in \{0,1\} & & & & & \end{array} \end{array}$$

where the objective function (1) is used to minimize the total tour distance, the entering constraints (2) indicate that the traveler can only travel to city j once, and the departure constraints (3) indicate that the salesman can only depart from city i once. Constraints (2) and (3) ensure that the traveler travels to each city only once, but do not eliminate the possibility of any subtours. However, the elimination constants (4) prevent the formation of any subtours by the traveler.

## DFOA for TSP

### Basic FOA

Fruit flies can distinguish various aromas to identify food sources as much as 40 km away. Fruit flies can also identify food sources based on the flocking positions of other fruit flies. When a fruit fly forages for food, it flies randomly in search of a position with a particular odor. While foraging, a fly can send and receive information from its partners in order to compare the fitness and determine the optimum location. After a fly discovers a favorable location, it determines the fitness of the location via tasting. If the position no longer exists or the taste is 'biting', the fly will stop searching. The fly will then remain near the optimum region and send, receive, and compare information with its partners. Pan developed the original FOA based on this searching behavior [18].

The basic FOA consists of a unique initialization process and a maximum generation-based cycle of smelling, evaluating, and flocking. First, the control parameters of the FOA are defined, including the maximum number of generations and population size, and the position of the fruit fly swarm is initialized at random. Then, the FOA initiates the smelling, evaluation, and concentration processes.

During the smelling process, a population of fruit flies utilize osphresis to search for food sources near the fly fruit swarm location. After that, the population evaluates the location based on the fitness value or smell concentration of each fruit fly. When the best-known smell location is identified, the fruit fly swarm utilizes sight osphresis to move toward the source in a flocking process. The FOA repeats these three steps until reaching the maximum number of generations. Since the original FOA can only be applied to continuous optimization problems, it was modified herein to solve the TSP.

### Food Source Representation

In the continuous version of the FOA, each solution is represents a swarm location, or food source. Each fruit fly flies through the n-dimensional space by studying the historical potential location determined by the swarm population. For this reason, fruit flies tend to fly toward relatively good search regions during the foraging process [14]. Let the *X_axis* denote the swarm location. Then an individual fruit fly will search for a food source *X*_*i*_ = (*x*_*i*1_,*x*_i2_,…,*x*_*in*_) as follows:

$$X_{i}=X\_axis+RandomValue$$ (5)

**Fig 1:**
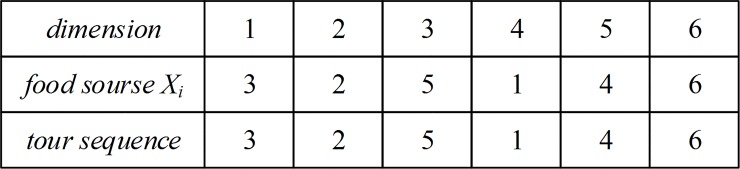
Food source and its corresponding tour.

In an instance of the TSP, a set of indices are given to identify sites. It is common to use these indices as cities' encoding order for the problem. Thus, in the direct encoding scheme, each dimension of the food source is used to represent an index of cities. Therefore, a food source identified by an individual fruit fly represents a sequence of the traveling route. An example of a feasible solution for a TSP with 6 cities is illustrated in Fig 1.

### Population Initialization

Rather than generating random tours that form the food location of an FOA's initial population, a tour construction heuristic, such as the nearest neighbor (NN) heuristic or other insertion heuristics, can be used. Since an entire population must be generated, the heuristic in use should be capable of creating distinct tours. For example, the closest neighbor heuristic allows for the construction of *n* distinct tours, where *n* is the number of cities, and each city can act as the starting point of the heuristic. In the simulation herein, when the population size is less than or equal to the number of cities, the initial population is created using a different starting point NN. Otherwise, the initial population is created via random permutation.

### Smelling Process

After a series of controlled initializations, fruit flies concentrate on the best-known swarm location. In addition, the surroundings, or the neighbors of the optimum swarm location, are explored. This is a basic explanation of the smelling search process. Based on this idea and the discrete particle swarm optimization of the TSP, a crossover operator ⊗ was introduced in order to rewrite Eq (5) for the generation of food sources in a basic FOA.

$$X_{i}^{t+1}=X_{i}^{t}⊗X\_axis$$ (6)

In Eq (6), $X_{i}^{t}$ represents the food source identified by fruit fly *i* at time step *t*, and *X_axis* is the optimum swarm location at time step *t*. Many studies concerning the crossover operator ⊗ have been conducted [1]. A simple two-point crossover approach was employed herein to determine how much information an individual fruit fly gathers from the swarm. This approach was selected due to its relatively high efficiency and effectiveness in large-scale problems compared to the PMX, OX, CX, and ERX approaches. The following steps were included in the crossover procedure:

**Step 1**. For the best-known food source *X_axis* identified by fruit flies, randomly generate two crossover positions *c*_1_ and *c*_2_ (*c*_1_ ≠ *c*_2_). Then, for the food source $X_{i}^{t}$ found by fruit fly *i* at time step *t*, delete the city indices in $X_{i}^{t}$ that are equivalent to the indices from position *c*_1_ to *c*_2_ in *X_axis*, the new incomplete food source $X_{i}^{t+1}$ is produced.

**Step 2**. In the deleted cities’ positions and the tail of $X_{i}^{t+1}$, suppose that $g_{{}_{j}}^{+}=L(X_{i}^{t})-L(X_{i}^{t+1})$ is the gain from inserting the segment from position *c*_1_ to *c*_2_ in *X_axis* into position *j*, and that $g_{{}_{j}}^{-}$ is the gain from inversely inserting the segment. If a position with a maximum of *g*_*j*_ exists and *g*_*j*_ ≥ 0, i.e. the tour length can be shortened, then insert the segment from position *c*_1_ to *c*_2_ in *X_axis*. Otherwise, restore $X_{i}^{t}$, such that $X_{i}^{t+1}$ is equivalent to $X_{i}^{t}$.

**Fig 2:**
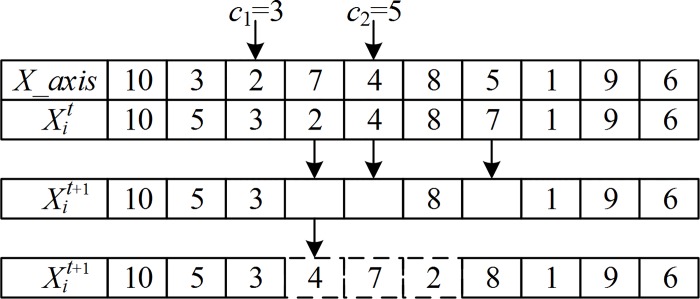
Crossover for the TSP.

An example of the individual fruit fly crossover with the best-known swarm location is illustrated in Fig 2. First, two crossover positions *c*_1_ = 3 and *c*_2_ = 5 are generated randomly in *X_axis*. Since the city indices 2, 7, and 4 in $X_{i}^{t}$ are equivalent to the indices from position *c*_1_ to *c*_2_ in *X_axis*, they are deleted from $X_{i}^{t}$. Thus, three alternative positions which can insert the segment (2,7,4) or its inverse (4,7,2) remain in $X_{i}^{t+1}$, including the places between 3 and 8, 8 and 1, and the place behind city 6. Suppose that the gain $g_{{}_{1}}^{+}$ between cities 3 and 8 is 60, where $g_{{}_{1}}^{-}$ is 65, the gain $g_{{}_{2}}^{+}$ between cities 8 and 1 is 55, where $g_{{}_{2}}^{-}$ is 50, and the gain $g_{{}_{3}}^{+}$ behind city 6 is 40, where $g_{{}_{3}}^{-}$ is 45. As $\max\;\{g_{1}^{+},g_{1}^{-},g_{2}^{+},g_{2}^{-},g_{3}^{+},g_{3}^{-}\}=g_{1}^{-}$, then, the first location is selected, and segment (4,7,2) is inserted.

When the doubly connection list is applied to the tour, the crossover operator can be computed using a constant time complexity, and the total complexity of the fruit fly swarm can be expressed as *O*(*n*).

Similar to the basic FOA, the population is evaluated with the DFOA by computing the smell concentration value of each fruit fly. However, in a TSP, the minimum tour length is considered best, and the smell concentration judgement value *Smell*_*i*_ is computed directly using the tour length of $X_{i}^{t}$. Then, the fruit fly with the best smell concentration is identified.

$$\left[Smell_{best}index_{best}\right]=min\left(Smell\right)$$ (7)

If the local *best Smell* value is less than the global *best Smell* value, the fruit fly group will retain the minimum concentration value *Smell*_*best*_ as $Smell_{best}^{g}$ *Smell* and save the *index*_*best*_ fruit fly as *X_axis*, or the optimum swarm location. At this time, the fruit fly swarm will use sight to fly toward that position.

However, if the value of *Smell*_*best*_ is not the global optimum, the basic FOA will repeat the smelling process on the last *X_axis*, and the solution can easily decay into local optimum. Therefore, it is necessary to include the not-so-good locations in further explorations.

### Tasting Process

In order to enhance the convergence speed of the DFOA, an edge intersection elimination (EXE) operator that accounts for the characteristics of the current best tour was introduced into the fruit fly tasting process, where edge-edge intersections in the current tour were considered reflective of improvement.

**Theorem 1.** *In a 2-dimensional Euclidean TSP*, *an optimal tour includes no edge-edge intersections*.

**Fig 3:**
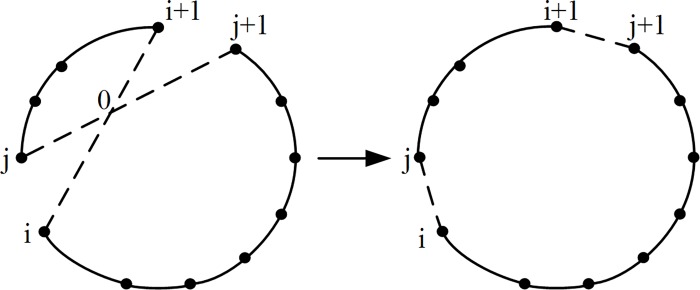
Edge intersection elimination exchange.

**Proof.** Let *X* = (…,*i*,*i* + 1,..,*j*,*j* + 1,…) be an optimal trip, where *L*(*X*) denotes the total cycle length. Assume that an edge (*i*,*i*+1) with a cross edge (*j*,*j*+1) and intersection point of 0 exists, as shown in Fig 3. If *j* is exchanged with *i* + 1 and the order of the indices between *j* and *i* + 1 are reversed, a new tour *X*’ = (…,*i*,*j*,..,*i* + 1,*j* + 1,…) can be obtained. If *X* is an optimal tour, then *L*(*X*') ≥ *L*(*X*). Therefore, the calculation of the total tour length *L*(*X*) contains *d*_i,i+1_ and *d*_j,j+1_, and the total tour length *L*(*X '*) includes *d*_i,j_ and *d*_i+1,j+1_. According to Fig 3,

$$d_{i,i+1}+d_{j,j+1}=d_{i,0}+d_{0}{}_{,i+1}+d_{j,0}+d_{0}{}_{,j+1}=d_{i,0}+d_{j,0}+d_{0,i+1}+d_{0,j+1}.$$

where *d*_i,0_+ *d*_j,0_> *d*_i,j_ and *d*_0,i+1_+*d*_0,j+1_>*d*_i+1,j+1_.

Thus, *d*_i,i+1_+*d*_j,j+1_>*d*_i,j_+*d*_i+1,j+1_. Since the remaining cumulative distances of *L*(*X*) are equal to *L*(*X '*),

L(X')<L(X),

which is a contradiction.

However, assume that two edges (*p*_1_,*p*_2_) and (*q*_1_,*q*_2_) exist. In a 2-dimensional Euclidean TSP, if the bounded rectangle of (*p*_1_,*p*_2_) does not cross the bounded rectangle of (*q*_1_,*q*_2_), (*p*_1_,*p*_2_) and (*q*_1_,*q*_2_) do not intersect. Otherwise, the following intersection judgment can be used:

1. If (*p*_1_,*p*_2_) intersects (*q*_1_,*q*_2_), then the vector products can be expressed as (*p*_1_-*q*_1_)×(*q*_2_-*q*_1_)×(*q*_2_-*q*_1_) ×(*p*_2_-*q*_1_)≥0.
2. If (*q*_1_,*q*_2_) intersects (*p*_1_,*p*_2_), then the vector products can be expressed as (*q*_1_-*p*_1_)×(*p*_2_-*p*_1_)×(*p*_2_-*p*_1_) ×(*q*_2_-*p*_1_)≥0.

Therefore, according to the above theory and intersection judgment rules, the steps of the EXE operator are as follows:

**Step 1.** From the current tour X, select the city by index *i*.
**Step 2.** From the current tour X, select the city by index *j* = *i+*2.
**Step 3.** If edge(*i*,*i*+1) intersects edge(*j*,*j*+1), then exchange *j* with *i*+1 and reverse the order of the indices between *j* and *i*+1 in *X*.
**Step 4**. If *i* = = 1 and *j*≤n-1, or *i*≠1 and *j*≤n, define *j* = *j*+1 and return to step 2.
**Step 5**. If *i*≤n-2, define *i* = *i*+1 and return to step 1.

In order to eliminate edge intersections, $C_{n}^{2}-n=n\left(n-3\right)/2$ edges must be examined for possible intersections with the given tour *X*. Therefore, the worst possible time complexity of the EXE operator can be written as *O*(*n*^2^).

**Fig 4:**
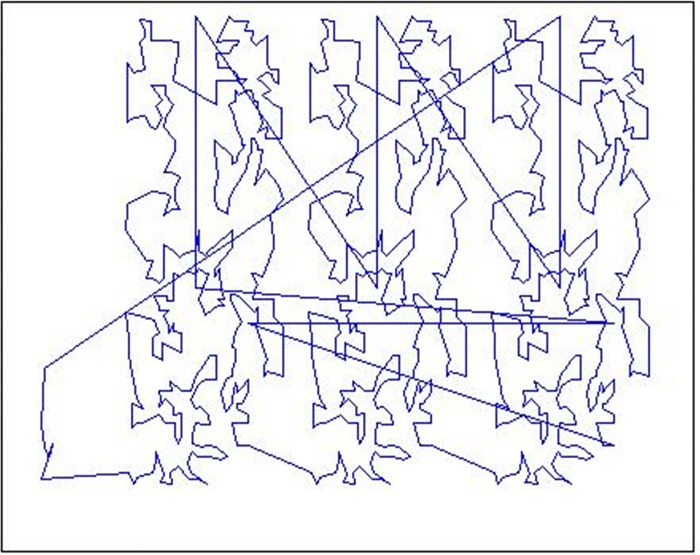
An initial tour for pr1002.

**Fig 5:**
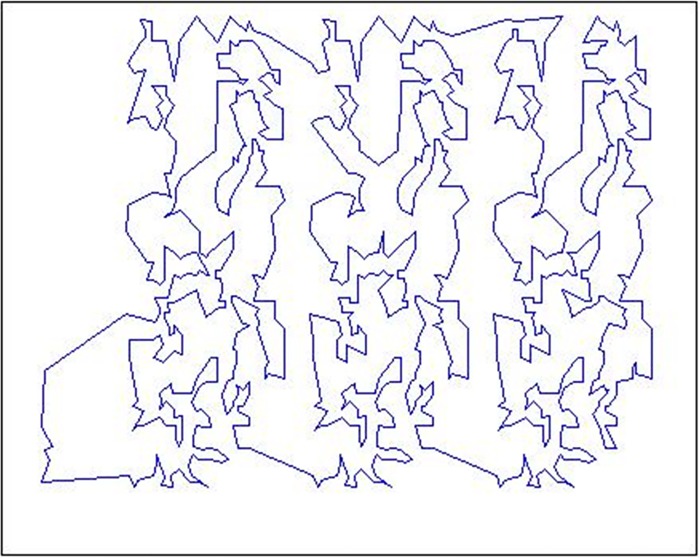
An EXE improved tour for pr1002.

An example of the EXE operator is illustrated from Fig 4 to Fig 5. Fig 4 shows the initial tour for TSPLIB problem pr1002 which is sequenced by city numbers, its tour length is 349438. It’s obvious that there are some edge-edge intersections in the current tour. Fig 5 shows the improved tour after carrying out the EXE operator, there are no edge-edge intersections, and its tour length is shortened into 280797. The changed gap is 19.64%.

### DFOA Procedures

**Fig 6:**
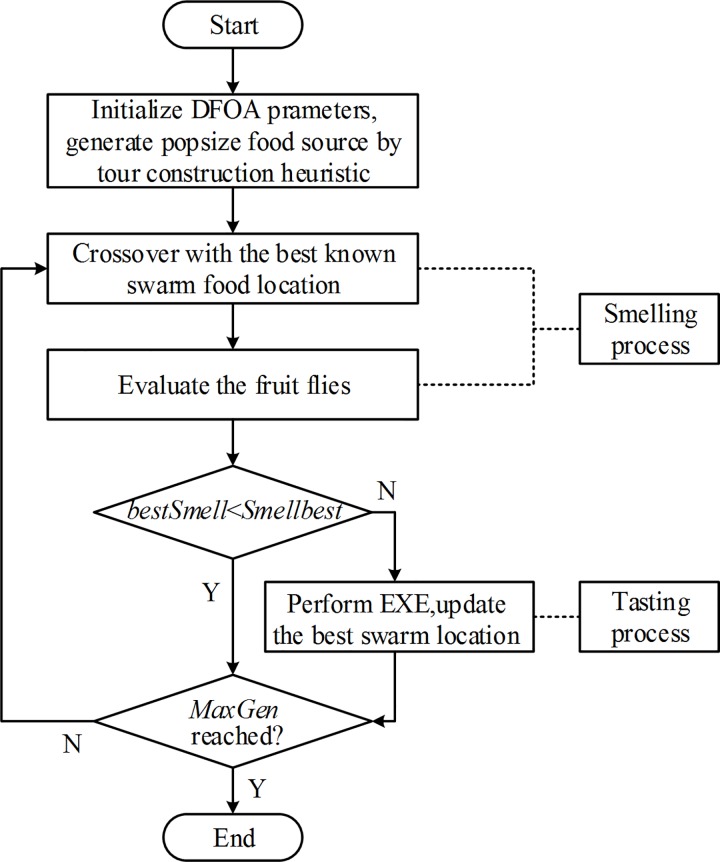
DFOA flowchart.

A flowchart of the DFOA is displayed in Fig 6.

As shown in Fig 6, other than the initialization process, the DFOA primarily involves two procedures. In the smelling procedure, the neighbors of the best-known swarm food location are exploited. In the tasting procedure, the neighbors of the non-optimum swarm food location are further investigated with the EXE operator, and the current best swarm food location is updated. Because both of exploitation and exploration are considered in the proposed DFOA, it was expected to yield satisfactory results when applied to the TSP.

## Numerical Testing Results and Comparisons

A set of benchmark TSP cases (available at the Web site http://www.iwr.uni-heidelberg.de/groups/comopt/software/TSPLIB95/) are used for numerical tests in literature. Those selective instances (S1 File) were also used for the numerical tests in this paper. The proposed DFOA was coded using ANSI C and executed with the GNU gcc compiler (version 4.83) on an Intel Machine with Core2 TM, a 2.3 GHz processor, and 4GB RAM. The DFOATSP program is provided in S2 File. The proposed DFOA included only two fundamental parameters, the population size (*N*), and maximum number of generations (*MaxGen*), which were defined as *N* = 5 and *MaxGen* = 100 herein, respectively. The DFOA, Parallel Hybrid Genetic Algorithm (PHGA) [42], and PSO were applied to small, middle, and large instances 20 times each using the same parameters. The results, including the reported optimal tour length (Optimum), average tour length (Mean), standard deviation (SD), best tour length (Best), and average CPU running time (CPU), are provided in Tables 1–3. Due to its large computation load and extended CPU running time, the PSO did not yield results when applied to the large instances.

**Table 1:** Comparison of the DFOA, PHGA, and PSO on small-scale TSP instances after 20 repetitions.

TSP instances		PHGA		PSO		DFOA			
Name	Optimum	Mean	SD	Mean	SD	Best	Mean	SD	CPU(s)
D493	35002	35032.7	43.9	36012.3	140.5	35002	35010.9	15.4	5.0
U574	36905	36983.2	91.3	37113.9	157.2	36905	36933.9	30.9	2.6
Pcb442	50778	50838.2	89.7	50923.3	213.7	50778	50841.5	65.4	3.0
Rat575	6773	6779.5	10.1	6798.6	40.3	6773	6777.3	2.4	2.1
Ali535	202310	202389.1	91.5	202350.8	256.9	**202308**	202323.6	25.3	2.5

**Table 2:** Comparison of the DFOA, PHGA, and PSO on medium-scale TSP instances after 20 repetitions.

TSP instances		PHGA		PSO		DFOA			
Name	Optimum	Mean	SD	Mean	SD	Best	Mean	SD	CPU(s)
Pr1002	259045	259246.7	230.6	259323.5	278.8	259045	259144.1	117.4	2.0
Fl1400	20127	20189.4	89.3	20235.3	145.9	20127	20138.9	12.9	3.2
vm1748	336556	336644.3	110.3	336778.1	289.4	336556	336570.8	15.4	9.0
Rl1304	252948	253100.5	79.8	253178.3	167.3	252948	252960.3	13.5	3.0
Pcb1173	56892	56983.6	120.5	57113.2	158.9	56892	56903.6	15.3	2.5

**Table 3:** Comparison of the DFOA and PHGA on large-scale TSP instances after 20 repetitions.

TSP instances		PHGA		DFOA			
Name	Optimum	Mean	SD	Best	Mean	SD	CPU(s)
Rl11849	923132	923360.3	302.4	923132	923250.3	213.4	260
Rl5915	565530	565623.7	113.3	565530	565613.4	122.9	52
Fl3795	28772	28904.3	167.9	28772	28890.2	135.4	116
D2103	80330	80453.6	156.3	80330	80422	113.5	123
U2319	234256	234396.1	157.8	234256	234273.0	29.3	32

As shown in Tables 1–3, the DFOA yielded smaller average tour length values than the other two algorithms, indicating that the proposed DFOA was more effective when applied to the TSP. As stated in the population and number of PHGA evolution generations can be increased to obtain better results. However, these measures would increase the computational load and time. Therefore, the proposed DFOA required less CPU time than the PSO and PHGA.

## Conclusions

In this study, a novel discrete fruit fly optimization algorithm was applied to the traveling salesman problem (TSP). An effective crossover operator was developed in order to allow the fruit fly group to search the neighbors of the best-known swarm location. Tasting and smelling processes were introduced into the algorithm. In addition, an edge intersection elimination operator was incorporated into the DFOA in order to improve the neighbors of the non-optimum food location. According to the results of the computational tests and comparisons, the proposed DFOA yielded better answers for all of the cases with less computational effort. This research not only provided a TSP with a powerful solution algorithm, but also realized the application of a DFOA to a discrete field. The proposed DFOA is a population-based parallel algorithm with few required simple search frameworks and control parameters. A number of local search operators or knowledge-based principles can be easily implanted into the framework of the proposed DFOA.

Therefore, future work could focus on the development of adaptive algorithms with parameter learning mechanisms and the implementation of other problem-specific features that could improve the performance of the DFOA. In addition, the proposed DFOA could be applied to other variations of the TSP, such as fixed edges are listed that are required to appear in each solution to the problem, Hamiltonian cycle or path problem, Capacitated vehicle routing problem etc. Furthermore, while the proposed encoding schema is hard to carry out crossover, an effective solution representation schema, which is suitable for crossover and inheriting the properties from parental tour, can be designed in a future work. Moreover, new candidate sets generation mechanisms, not only the NN, but a good estimate of the edges’ chances of belonging to an optimal tour, and more effective local search methods can be used in a future work.

## Supporting Information

S1 File Testing TSP instances. (ZIP)

S2 File DFOATSP program. (ZIP)
