# Supplementary material for: A Discrete Fruit Fly Optimization Algorithm for the Traveling Salesman Problem
Source: PLoS One. 2016 Nov 3;11(11):e0165804. doi: 10.1371/journal.pone.0165804 (PMC5094794; doi:10.1371/journal.pone.0165804)
Supplement: S2 File — (ZIP) [file pone.0165804.s002.zip › DFOATSP/ExeOPT.docx]

void ExeOPT(int *Route)

{

int ahead,i,i1,i2,index;

int j,j1,j2,last,limit,next;

int nCrossed;

/* The edges in the current solution are kept in the array pSTR, That is, (i,pSTR[i])

is an edge in the current solution,incident on node I*/

pSTR[0] = 0;

for(i=1;i<=Dimension-1;i++){

pSTR[Route[i]] = Route[i+1];

}

/* the last tour city succeed by the first tour city*/

pSTR[Route[Dimension]] = Route[1];

/*Edge Intersection Eliminate*/

/* do{*/

nCrossed = 0;

i1=1;

for(i=1;i<=Dimension-2;i++){

/*last case is only when i=1*/

if(i==1){

limit = Dimension-1;

}

else{

limit = Dimension;

}

i2 = pSTR[i1];

j1 = pSTR[i2];

/* the rest n-2 edges*/

for(j=i+2;j<=limit;j++){

j2 = pSTR[j1];

if(IsEdgeIntersection(i1,i2,j1,j2)==1){

pSTR[i1] = j1;

next = i2;

last = j2;

/* Reverse appropriate links*/

do{

ahead = pSTR[next];

pSTR[next]= last;

last = next;

next = ahead;

}while(next!=j2);

/* Route is now shorter*/

nCrossed = 1;

i2 =pSTR[i1];

j1=pSTR[i2];

}

else{

j1 = j2;

}

}

i1 = i2;

}

/* }while(nCrossed>0);*/

/*restore the route*/

index = 1;

for(i=1;i<=Dimension;i++){

Route[i] = index;

index = pSTR[index];

}

}

static int IsEdgeIntersection(int p1,int p2,int q1,int q2)

{

double v1,v2,v3,v4;

double p1x,p1y,p2x,p2y,q1x,q1y,q2x,q2y;

p1x=NodeSet[p1].X;

p1y=NodeSet[p1].Y;

p2x=NodeSet[p2].X;

p2y=NodeSet[p2].Y;

q1x=NodeSet[q1].X;

q1y=NodeSet[q1].Y;

q2x=NodeSet[q2].X;

q2y=NodeSet[q2].Y;

/*fast excluding*/

if(max(p1x,p2x)<min(q1x,q2x)||min(p1y,p2y)>max(q1y,q2y)){

return 0;

}

if(max(q1x,q2x)<min(p1x,p2x)||min(q1y,q2y)>max(p1y,p2y)){

return 0;

}

/*p1p2×p1q2*/

v1=(p2x-p1x)*(q2y-p1y) - (p2y-p1y)*(q2x-p1x);

/*p1p2×p1q1*/

v2=(p2x-p1x)*(q1y-p1y) - (p2y-p1y)*(q1x-p1x);

if(v1*v2>0){

return 0;

}

/*q1q2×q1p2*/

v3=(q2x-q1x)*(p2y-q1y) - (q2y-q1y)*(p2x-q1x);

/*q1q2×q1p1*/

v4=(q2x-q1x)*(p1y-q1y) - (q2y-q1y)*(p1x-q1x);

if(v3*v4>0){

return 0;

}

return 1;

}
