# Supplementary material for: A Discrete Fruit Fly Optimization Algorithm for the Traveling Salesman Problem
Source: PLoS One. 2016 Nov 3;11(11):e0165804. doi: 10.1371/journal.pone.0165804 (PMC5094794; doi:10.1371/journal.pone.0165804)
Supplement: S2 File — (ZIP) [file pone.0165804.s002.zip › DFOATSP/User Guide.docx]

# User Guide

On a Windows machine execute the following commands:

Unzip DFOATSP.zip

Run command line

cd the directory of the whole unzipped files.

Run the programming by typing:

DFOATSP pr1002.par


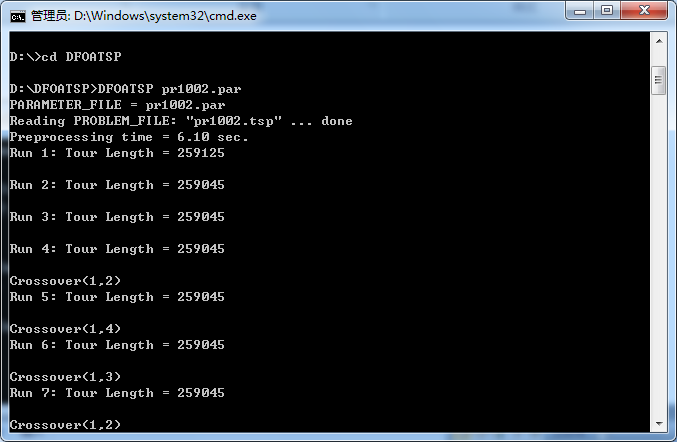


You can also run the program by just double click DFOATSP.exe, but you have to input the parameter file (*.par) after the prompt “PARAMETER_FILE =”.
